# On the potential application of polar and temperate marine microalgae for EPA and DHA production

**DOI:** 10.1186/2191-0855-3-26

**Published:** 2013-05-14

**Authors:** Peter Boelen, Roechama van Dijk, Jaap S Sinninghe Damsté, W Irene C Rijpstra, Anita GJ Buma

**Affiliations:** 1 Department of Ocean Ecosystems, Energy and Sustainability Research Institute, University of Groningen, Nijenborgh 7, Groningen, 9747 AG, The Netherlands; 2 Department of Marine Organic Biogeochemistry, Royal Netherlands Institute for Sea Research (NIOZ), Landsdiep 4, 1797 SZ, ’t Horntje, Texel, The Netherlands

**Keywords:** Eicosapentaenoic acid, Docosahexaenoic acid, Thalassiosira weissflogii, Chaetoceros brevis, Fibrocapsa japonica, Emiliania huxleyi, Pyramimonas sp.

## Abstract

Long chain polyunsaturated fatty acids (LC-PUFAs) such as eicosapentaenoic acid (EPA) and docosahexaenoic acid (DHA) are considered essential omega-3 fatty acids in human nutrition. In marine microalgae EPA and/or DHA are allegedly involved in the regulation of membrane fluidity and thylakoid membrane functioning. The cellular content of EPA and DHA may therefore be enhanced at low temperature and irradiance conditions. As a result, polar and cold temperate marine microalgal species might potentially be suitable candidates for commercial EPA and DHA production, given their adaptation to low temperature and irradiance habitats.

In the present study we investigated inter- and intraspecific EPA and DHA variability in five polar and (cold) temperate microalgae. Intraspecific EPA and DHA content did not vary significantly in an Antarctic (*Chaetoceros brevis*) and a temperate (*Thalassiosira weissflogii*) centric diatom after acclimation to a range of irradiance levels at two temperatures. Interspecific variability was investigated for two Antarctic *(Chaetoceros brevis* and *Pyramimonas* sp. (*Prasinophyceae*)) and three cold-temperate species (*Thalassiosira weissflogii, Emiliania huxleyi* (*Prymnesiophyceae*) and *Fibrocapsa japonica* (*Raphidophyceae*)) during exponential growth. Interspecific variability was shown to be much more important than intraspecific variability. Highest relative and absolute levels of DHA were measured in the prymnesiophyte *E. huxleyi* and the prasinophyte *Pyramimonas* sp., while levels of EPA were high in the raphidophyte *F. japonica* and the diatoms *C. brevis* and *T. weissflogii*. Yet, no significant differences in LC-PUFA content were found between polar and cold-temperate species. Also, EPA and DHA production rates varied strongly between species. Highest EPA production rate (174 μg L^-1^ day^-1^) was found in the Antarctic diatom *Chaetoceros brevis*, while DHA production was highest in the cold-temperate prymnesiophyte *Emiliania huxleyi* (164 μg L^-1^ day^-1^). We show that, following careful species selection, effective mass cultivation of marine microalgae for EPA and DHA production may be possible under low temperature and irradiance conditions.

## Introduction

Marine microalgae are key organisms in the production of long chain (between 14 and 22 carbon atoms) polyunsaturated fatty acids (LC-PUFAs) in marine food webs (Harwood and Jones 1989;Guschina and Harwood 2006). The most common LC-PUFAs found in marine microalgae are the omega-3 fatty acids eicosapentaenoic acid (EPA, 20:5n3) and docosahexaenoic acid (DHA, 22:6n3), and, to a lesser extent, the omega-6 fatty acid arachidonic acid (AA, 20:4n6). Both EPA and DHA are particularly found in taxa belonging to the Chromalveolata, such as diatoms, dinoflagellates and prymnesiophytes (Volkman et al. 1989;Tonon et al. 2002;Mansour et al. 2005).

EPA and DHA are considered essential elements in human nutrition. There are strong indications that LC-PUFAs play an important role as membrane components involved in our neural system (Uauy et al. 2000). Furthermore, EPA and/or DHA function as hormone precursors and are thought to play a role in the immune system (Calder 1997;Uauy et al. 2000). Humans are able to synthesize EPA and DHA, but only at very low rates (Emken et al. 1994). EPA and DHA are formed from alpha-linoleic acid (ALA, 18:3n3), which is only present in substantial amounts in linseed oil and therefore almost completely missing in our diet (Ollis et al. 1999). As a result of this, there is a growing market for EPA- and DHA-enriched products (e.g. in bread, margarine). Although microalgae are the primary producers of EPA and DHA in marine food webs, until now their principal source for human nutrition is marine fish oil (Sinclair 2000). To meet the needs of this market without further exploiting the world’s fish stocks it is necessary to search for alternatives. Culturing microalgae is an economically profitable option and, moreover, due to their CO_2_ uptake, environmentally favorable. In addition, the production costs of DHA or EPA from cultured algae is potentially equal to the cost of producing EPA from fish oil (Milledge 2011). However, the efficiency should be further increased by selecting EPA- and/or DHA-rich species while at the same time optimizing mass cultivation and harvest conditions.

For long chain PUFAs, such as EPA and DHA, a major role in maintaining cell membrane fluidity is assumed (Nichols et al. 1993;Nishida and Murata 1996). For this reason, having elevated relative EPA and/or DHA levels could form a critical metabolic requirement for survival in polar regions, where microalgae typically live at temperatures near their freezing point (Thomas and Dieckmann 2002). Furthermore, EPA or other long chain PUFAs may play a role in the functioning of the thylakoid membrane and thus they are essential for photosynthesis (Kates and Volcani 1966;Cohen et al. 1988). At higher irradiance algae become less photosynthetically efficient and thus less thylakoid membranes are required. As a result, LC-PUFA content could be lower in high light-acclimated algae (Harwood and Jones 1989). In other words, cold-adapted polar microalgae might be good candidates for mass EPA and DHA production, since they can exhibit high growth rates under low temperature and irradiance conditions. Nevertheless earlier studies (e.g. Grima et al. 1995;Otero et al. 1997;Carvalho and Malcata 2005;Huerlimann et al. 2010) addressing the potential for EPA/DHA mass production focused primarily on warm (temperate) species. Although, in general, cultivation under high irradiance and temperature conditions will lead to higher growth rates (Raven and Geider 1988;Geider et al. 1998), this may not be optimal for LC-PUFA productivity. With respect to the intraspecific effect of irradiance and temperature earlier studies showed contradictive results. For example, the effect of irradiance was found to be strongly species-specific. In some of the studies the expected effect was found (Thompson et al. 1990;Guedes et al. 2010;Leu et al. 2010), but differences were often small while sometimes no or opposite irradiance effects were found (Chrismadha and Borowitzka 1994;Tzovenis et al. 1997;Carvalho and Malcata 2005;Zhukova 2007;Guiheneuf et al. 2009). Similarly, with respect to temperature, no consistent results were found (Thompson et al. 1992;Jiang and Gao, 2004;Teoh et al. 2004;Chen et al. 2008). Other factors influencing PUFA content suggested are CO_2_ concentration, nutrient availability, growth phase and day-night rhythm (Mayzaud et al. 1990;Yonmanitchai and Ward 1991;Zhukova 2004;Meiser et al. 2004;Lv et al. 2010). Besides intraspecific EPA and DHA variability, it is known that EPA and DHA content shows significant differences between and within algal classes. Each algal class has roughly its own fatty acid composition and the EPA-DHA content between algal classes is highly variable (Brown 2002;Guschina and Harwood 2006;Lv et al. 2010). Furthermore, as mentioned above, polar species are expected to sustain a higher EPA and DHA content, since their habitat is characterized by lower average irradiance and temperature levels (van Leeuwe 1997).

The aim of this study was first of all to get a better insight into the intraspecific variability in EPA and DHA content in response to irradiance and temperature. To investigate this, cellular EPA and DHA content of a polar and a temperate diatom was followed after acclimation to a range of irradiance levels at two temperatures. Secondly, the interspecific EPA and DHA variability between and within polar and temperate species was investigated. To this end, EPA and DHA content of two polar and three temperate species, cultured at equivalent growth conditions were investigated. So far, no comparative analysis between temperate and polar algal species had been carried out. In addition, many earlier studies reporting on EPA and DHA variability in polar species focused on relative variability, rather than on absolute cellular production rates. To be able to measure true interspecific differences, taking into account differences in total fatty acid content relative to algal biomass, the absolute amounts of EPA and DHA were determined, using biovolume as biomass unit.

## Materials and methods

### Pre-experimental cultivation

Two polar and three temperate species were selected representing different taxonomic groups (Table 1). Cultures of the polar species were maintained at 4°C and the temperate species at 16°C, except for *Fibrocapsa japonica*, which was kept at 18°C. *Fibrocapsa japonica* was cultured at an irradiance of 35 μmol photons m^-2^ s^-1^. For the other species the pre-experimental culture irradiance was approximately 10 μmol photons m^-2^ s^-1^. Before and during experimentation all species were subjected to a light–dark cycle of 16:8. Cultures were grown in F2 medium (Guillard and Ryther 1962) based on filtered natural sea water originating from the Canary Basin. For *F. japonica* the salinity of the medium was set at 25 PSU and for the other species at 35 PSU.

**Table 1:** Details of investigated species and culturing conditions

				**Culture conditions**	
**Strain**	**Class**	**Strain#**	**Biovolume (μm**^**3**^**)**	**Temperature (°C)**	**Irradiance (μmol photons m**^**-2 **^**s**^**-1**^**)**
*Chaetoceros brevis*	*Bacillariophyceae*	CCMP 163	351	3 and 7	10, 25, 75 and 150
*Thalassiosira weissflogii*	*Bacillariophyceae*	CCMP 1049	2298	16 and 20	10, 25, 75 and 150
*Pyramimonas* sp.	*Prasinophyceae*	RuG collection	95	3	75
*Emiliania huxleyi*	*Prymnesiophyceae*	RuG collection	35	16	75
*Fibrocapsa japonica*	*Raphidophyceae*	RuG collection	4400	16	75

### Experimental setup

Two sets of experiments were performed. Intraspecific variability in LC-PUFA content was investigated in a polar (*Chaetoceros brevis*) and a temperate (*Thalassiosira weissflogii*) centric diatom. *Chaetoceros brevis* was cultured at 3 and 7°C and *T. weissflogii* at 16°C and 20°C, both at irradiances of 10, 25, 75 and 150 μmol photons m^-2^ s^-1^. The irradiance levels were based on earlier experiments on Antarctic and cold temperate phytoplankton (van de Poll et al. 2007;Boelen et al. 2011) and simulate limiting and saturating culture conditions in a stable water column. Interspecific LC-PUFA variability was investigated for two polar (*C. brevis* and *Pyramimonas* sp.) and three temperate (*F. japonica*, *T. weissflogii* and *Emiliania huxleyi*) species. For the latter set of experiments all species were cultured at the same irradiance (75 μmol photons m^-2^ s^-1^) at 16°C for the temperate species and 3°C for the polar species. Algae were cultured in duplicate in Fernbach or Erlenmeyer flasks with a working volume of approximately 0.5 L. The experiments were set up in water baths, connected to Neslab cryostats, to assure accurate temperature control (± 0.5°C). Before samples were collected for fatty acid and pigment analysis (see below), cultures were acclimated to the different temperatures and irradiance levels in semi-continuous batch mode for at least one week; fast growing cultures were diluted with fresh medium to avoid nutrient limitation. Sampling took place during exponential growth in the middle of the light period. Cell counts were done regularly to establish growth rates. Specific growth rates (μ) were calculated from linear regressions of the natural log of cell numbers versus time. Cell biovolumes were calculated from microscopic images as described below.

### Analytical procedures

Cell numbers were determined using a Coulter XL-MCL flow cytometer (Beckman Coulter, Miami, FL, USA) as described by van de Poll et al. (2005).

To determine the pigment composition, samples of cultures (30 mL) were filtered through GF/F filters (25 mm), immediately frozen in liquid nitrogen and stored at −80°C until further analysis. The filters were freeze-dried (48 h) and extracted with 90% (aqueous) acetone for 48h at 4°C. Pigments were separated and quantified on a Waters HPLC system (model 2690) equipped with a 996 photodiode array detector and a C_18_ 5 μm DeltaPak reverse-phase column as described by van Leeuwe et al. (2006).

For fatty acid analysis, 100 mL of the culture were filtered through pre-combusted GF/F (25 mm) filters, frozen in liquid nitrogen and stored at −80°C until further analysis using gas chromatography (GC) and gas chromatography–mass spectrometry (GC-MS). The procedure is modified from the method described by Klein Breteler et al. (2004). The samples were freeze-dried for 48 h and a known amount of nonadecanoic acid (C19:0, Fluka) was added as an internal standard. The samples were saponified by reflux (1 h) with 1 N KOH-MeOH (96%). After acidifying with 2N HCl-MeOH (1:1) to a pH of 4 the filter was removed by centrifugation and bidistilled water was added to the supernatant in a ratio equal to MeOH. Fatty acids were extracted from this mixture with dichloromethane (DCM) (3×). The DCM extract was dried over Na_2_SO_4_ and methylated with diazomethane. The non-polar fatty acid methyl esters were separated from the polar compounds over a small Al_2_O_3_ column using DCM as eluent and analyzed on a Hewlett–Packard 6890 gas chromatograph equipped with a fused silica capillary column (50m × 0.32mm) coated with CP Sil-5 CB (film thickness 0.12 μm). Helium was used as carrier gas. The oven thermal gradient rose from an initial 70°C to 130°C at 20°C min^-1^ and then to a final temperature of 320°C increasing 4°C min^-1^, which was maintained for 10 min. Selected samples were also analyzed by GC-MS. GC–MS was performed with a Hewlett–Packard 5890 gas chromatograph interfaced with a VG Autospec Ultima mass spectrometer operating at 70 eV, with a mass range of m/z 50–800 and a cycle time of 1.7 s (resolution 1000). The gas chromatograph was equipped with an on-column injection system and the same capillary column as described for GC. The carrier gas was helium. The temperature program was the same as described for GC. Long chain fatty acids (number of carbons ≥ 14) were identified from mass spectra and retention times and the double-bond positions were determined by comparison with those of PUFA No.1 standard mixture (Matreya). Quantification of fatty acids was done by integration of appropriate peak areas and using the known concentration of the added internal standard.

To calculate the biovolume of the algae, a sample of about 2 ml culture was analyzed using an inverted microscope. The sizes of 50 cells were measured and biovolume (Table 1) was calculated according to Hillebrand et al. (1999) assuming a cylinder for *C. brevis* and *T. weissflogii*, halve an ellipse for *Pyramimonas* sp., and a sphere for *Emiliania huxleyi*. The biovolume of *F. japonica* was taken from de Boer (2006).

EPA and DHA production rates (P_PUFA_ [μg L^-1^ day^-1^]) were calculated from specific growth rate (μ [day^-1^]), cellular EPA or DHA content (C_PUFA_ [μg cell^-1^]) and maximum cell density (N_m_ [cells L^-1^]) according to the equation P_PUFA_ = μ × C_PUFA_ × N_m_. Since maximum cell densities were not the same at all culture conditions, the calculations were based on averages of final cell numbers at harvest in cultures grown at standard irradiance conditions (75 μmol photons m^-2^ s^-1^).

### Statistical analysis

Significant differences between treatments were analyzed with a one-way analysis of variance (ANOVA) and were considered not significant at p > 0.05. Post-hoc tests (Tukey HSD) were performed to further specify differences.

## Results

### Effect of temperature and irradiance

**Figure 1:**
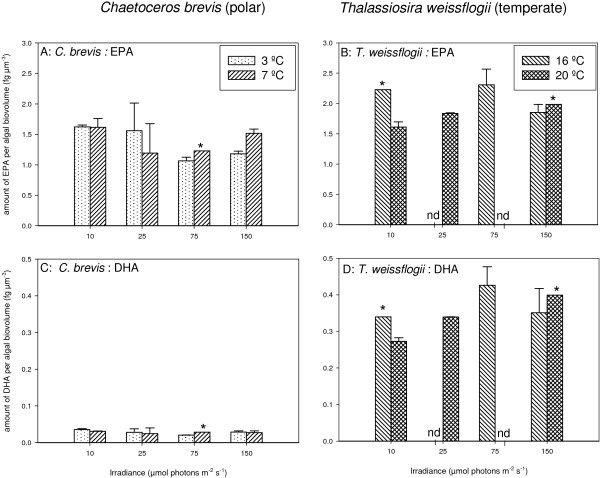
**EPA and DHA content (normalized to biovolume) of *C. brevis *(A,C) and *Th. weissflogii *(B,D) grown at two temperatures and four irradiance levels.** Values represent averages (± SD) based on two replicate cultures for each condition. nd = no data available. * no replicate.

For both investigated species, *C. brevis* and *T. weissflogii*, growth rates and chlorophyll *a* levels were clearly influenced by irradiance levels; growth rates increased at increasing irradiance levels, while chlorophyll *a* content per cell was significantly higher at low irradiance (Table 2). For the polar diatom *C. brevis* no significant effect of temperature on growth rate and chlorophyll *a* could be demonstrated. For *T. weissflogii* a small but significant effect of temperature on chlorophyll *a* content was found, being slightly higher at 16°C (standard culture temperature) compared to 20°C (Table 2). The effect of temperature on specific growth rate was not determined for this species. Since chlorophyll *a* levels were affected by irradiance and temperature, standard biovolume values were used as biomass units. No significant effect of temperature and irradiance on EPA and DHA content could be demonstrated for both species (Figure 1, Table 2).

**Table 2:** **Chlorophyll *a *per cell, specific growth rate (μ), EPA and DHA content (normalized to biovolume) of two polar ( *C. brevis *, *Pyramimonas *sp.) and three temperate ( *T. weissflogii *, *E. huxleyi *and *F. japonica*) microalgal species (mean value (± SD) of two replicate cultures)**

	**Temperature (°C)**	**Irradiance (μmol m**^**-2 **^**s**^**-1**^**)**	**μ (day**^**-1**^**)**	**Chloroph. *a *(pg cell**^**-1**^**)**	**EPA content (fg μm**^**-3**^**)**	**DHA content (fg μm**^**-3**^**)**
*Chaetoceros brevis*	3	10	0.22 ± 0.02	0.50 ± 0.03	1.62 ± 0.03	0.04 ± 0.00
	3	25	0.35 ± 0.02	0.39 ± 0.02	1.56 ± 0.45	0.03 ± 0.01
	**3**	**75**	**0.47 ± 0.01**	**0.15 ± 0.01**	**1.06 ± 0.06**	**0.02 ± 0.00**
	3	150	0.43 ± 0.02	0.15 ± 0.00	1.18 ± 0.05	0.03 ± 0.00
	7	10	0.24 ± 0.03	0.52 ± 0.06	1.61 ± 0.14	0.03 ± 0.00
	7	25	0.36 ± 0.06	0.44 ± 0.11	1.19 ± 0.48	0.02 ± 0.02
	7	75	0.41 ± 0.00	0.13 ± 0.01	1.23*	0.03*
	7	150	0.42 ± 0.02	0.13 ± 0.00	1.52 ± 0.07	0.03 ± 0.01
*Thalassiosira weissflogii*	16	10	0.29 ± 0.06	9.77 ± 0.03	2.23*	0.34*
	16	25	0.36 ± 0.01	9.84 ± 0.70	nd	nd
	**16**	**75**	**0.42 ± 0.08**	**4.50 ± 1.14**	**2.31 ± 0.26**	**0.43 ± 0.05**
	16	150	0.49 ± 0.09	3.05 ± 0.00	1.85 ± 0.13	0.35 ± 0.07
	20	10	0.31 ± 0.01	9.53 ± 0.06	1.61 ± 0.08	0.27 ± 0.01
	20	25	nd	8.40 ± 0.33	1.83 ± 0.01	0.34 ± 0.00
	20	75	nd	3.67 ± 0.38	nd	nd
	20	150	nd	2.93 ± 0.09	1.99*	0.40*
*Pyramimonas* sp.	**3**	**75**	**0.14 ± 0.03**	**1.54 ± 0.16**	**0**	**8.75 ± 0.28**
*Emiliania huxleyi*	**16**	**75**	**0.34 ± 0.08**	**0.18 ± 0.00**	0.89*	27.7*
*Fibrocapsa japonica*	**16**	**75**	**0.44 ± 0.04**	**15.99 ± 0.71**	**2.93 ± 0.30**	**0**

Standard culture conditions are indicated in bold. nd = no data available. * no replicate.

### Interspecific PUFA variability

The fatty acid composition showed large variability between species, cultured at standard irradiance (75 μmol photons m^-2^ s^-1^) and temperature (3ºC and 16°C for polar and temperate species, respectively) (Table 3). Relative amounts of long chain PUFAs, expressed as percent of total long chain fatty acids (number of carbons ≥ 14), varied between 34–62%. EPA and DHA content varied strongly between species. In *F. japonica* and the diatoms *C. brevis* and *T. weissflogii* high abundances (between 12–32%) of EPA were detected, while levels of DHA were low (< 3%). In contrast, *E. huxleyi* contained high relative proportions of DHA (21%) and minimal (< 1%) amounts of EPA. Other PUFAs present in substantial amounts were a 16:4 polyunsaturated fatty acid (*C. brevis* and *Pyramimonas* sp. only), alpha-linolenic acid (ALA; 18:3(n-3)) (*Pyramimonas* sp. and *E. huxleyi*). In *Pyramimonas* sp. also small amounts (3%) of docosapentaenoic acid (DPA; 22:5(n-3)) were found.

**Table 3:** **Fatty acid composition (% of total long chain fatty acid) of the five investigated species, cultivated at standard irradiance (75 μmol photons m**^**-2 **^**s**^**-1**^**) and temperature (polar species: 3°C, temperate species: 16°C) conditions**

***Fatty acid***	***Chaetoceros brevis***	***Thalassiosira weissflogii***	***Pyramimonas *sp.**	Emiliania huxleyi*	***Fibrocapsa japonica***
C14:0	7.6 ± 0.1	7.6 ± 0.4	-	20.2	22.1 ± 0.1
C15:0	-	1.3 ± 0.1	-	1.1	-
C16:0	7.9 ± 0.1	26.8 ± 0.3	23.2 ± 0.3	6.2	18.6 ± 0.2
C16:1	2.8 ± 0.0	-	1.5 ± 0.0	-	1.5 ± 0.1
C16:1n7	26.6 ± 0.1	30.2 ± 0.4	-	-	2.7 ± 0.8
C16:3	3.4 ± 0.1	11.9 ± 0.1	-	-	-
C16:4	14.2 ± 0.4	-	18.9 ± 1.0	-	-
C18:0	-	-	2.6 ± 1.8	-	1.4 ± 0.3
C18:1n5	-	-	2.8 ± 0.1	3.0	-
C18:1n7	-	-	5.5 ± 0.1	4.4	9.2 ± 3.0
C18:1n9	-	-	2.9 ± 0.1	17.1	10.9 ± 0.4
C18:2n6	-	-	3.8 ± 0.0	2.1	4.8 ± 0.0
C18:3n3	-	-	17.1 ± 0.4	8.9	2.2 ± 0.2
C18:4n3	5.6 ± 0.1	5.0 ± 0.3	5.9 ± 0.2	7.9	7.8 ± 2.1
C18:5n3	-	-	4.9 ± 0.2	8.4	-
C20:3	-	-	2.8 ± 0.1	-	-
C20:4n6	-	-	-	-	7.3 ± 0.6
C20:5n3 (EPA)	31.5 ± 0.4	14.6 ± 0.1	-	-	11.9 ± 1.5
C22:5n3	-	-	2.6 ± 0.1	-	-
C22:6n3 (DHA)	-	2.7 ± 0.0	5.8 ± 0.3	20.7	-

Values represent averages (± SD) based on two replicate cultures. * no replicate. – < 1%.

The highest absolute amount of DHA normalized to biovolume was measured in the prymnesiophyte *E. huxleyi* (27.7 fg DHA μm^-3^), followed by the Antarctic prasinophyte *Pyramimonas* sp. (8.75 fg DHA μm^-3^) (Table 2)*.* Relatively low DHA levels were observed for the Antarctic diatom *Chaetoceros brevis* (0.02 fg DHA μm^-3^), while DHA was undetectable in the raphidophyte *Fibrocapsa japonica* (Table 2). When normalized to biovolume, absolute EPA content was highest in *F. japonica* (2.93 fg μm^-3^). Lowest EPA levels were found in *Emiliania huxleyi* (0.89 fg EPA μm^-3^) while EPA was undetectable in *Pyramimonas* sp.. Overall, DHA levels showed the highest interspecific variability, varying two orders of magnitude on a biovolume basis (Table 2).

Although interspecific differences in EPA and DHA were substantial, no significant differences in relative and absolute amounts of EPA and DHA were observed between polar and temperate species.

### EPA and DHA production rates

Volumetric EPA and DHA productivity was calculated from cellular PUFA content, specific growth rates and maximal cell numbers (Table 4). Although in *F. japonica* EPA content per biovolume was highest (Table 2), total biomass production was higher in the Antarctic *C. brevis* leading to highest EPA productivity (at standard culture conditions circa 174 μg L^-1^ day^-1^). The small prymnesiophyte *E. huxleyi* showed the highest DHA productivity (164 μg L^-1^ d^-1^), even though growth rate and maximal biomass were relatively low in this species.

**Table 4:** **Calculated EPA and DHA production rates (μg L**^**-1 **^**day**^**-1**^**) during exponential growth for five species of microalgae based on specific growth rates and cellular PUFA content values from Table**2

	***Irradiance***	***EPA (μg L***^***-1 ***^***day***^***-1***^***)***		***DHA (μg L***^***-1 ***^***day***^***-1***^***)***	
	**(μmol m**^**-2 **^**s**^**-1**^**)**	**LT**	**HT**	**LT**	**HT**
*Chaetoceros brevis*	10	125 ± 14	138 ± 31	3 ± 0	3 ± 0
	25	193 ± 68	160 ± 84	4 ± 1	3 ± 2
	75	**174 ± 14**	176 ± 0.7	**3 ± 0**	4 ± 0
	150	175 ± 14	223 ± 20	4 ± 1	4 ± 1
*Thalassiosira weissflogii*	10	76 ± 17	60 ± 6	12 ± 3	10 ± 1
	75	**119 ± 36**	nd	**22 ± 7**	nd
	150	110 ± 27	nd	21 ± 7	nd
*Pyramimonas* sp.	75	**0**	nd	**5 ± 1**	nd
*Emiliania huxleyi*	75	**5 ± 1**	nd	**164 ± 41**	nd
*Fibrocapsa japonica*	75	**150 ± 30**	nd	**0**	nd

Standard culture conditions are indicated in bold. LT: culture temperature 3°C (polar species: *C. brevis*, *Pyramimonas* sp.) or 16°C (temperate species: *T. weissflogii*, *E. hux* and *F. japonica*). HT: culture temperature 7°C (polar species) or 20°C (temperate species). Cell densities at harvest: *C. brevis*: 9.9 × 10^5^ cells mL^-1^, *T. weissflogii*: 5.2 × 10^4^ cells mL^-1^, *Pyramimonas* sp.: 4.5 × 10^4^ cells mL^-1^, *E. hux* 5.0 × 10^5^ cells mL^-1^, and *F. japonica*: 2.6 × 10^4^ cells mL^-1^. Values represent averages ± standard deviation of two replicate cultures. nd = no data available.

For *C. brevis* and *T. weissflogii* EPA productivity was reduced at the lowest irradiance conditions, mainly due light limitation leading to reduced growth rates. For *C. brevis* no significant effect of temperature on EPA and DHA productivity could be demonstrated.

## Discussion

In this study we focused on EPA and DHA content and productivity in polar and (cold) temperate marine microalgae with an eye towards future high-latitude mass cultivation. With the method we have used for fatty acid analysis we did not distinguish between intracellular free fatty acids or those derived from triacylglycerols (TAGs), glycolipids or other lipid classes. This would compromise comparison with other studies focusing on fatty acids derived from extracted lipids (e.g. Lang et al. 2011), on LC-PUFA production in TAGs (Tonon et al. 2002) or on fatty acid profiles derived from specific lipid classes (Guckert et al. 1988).

The present study demonstrates large species-specific variability in EPA and DHA content, while irradiance and temperature showed relatively little to no effect. Highest relative and absolute levels of DHA were measured in the cold temperate prymnesiophyte *E. huxleyi* and the Antarctic prasinophyte *Pyramimonas* sp., while levels of EPA were high in the raphidophyte *F. japonica* and the diatoms *C. brevis* and *T. weissflogii*. Similar relative EPA and DHA compositions were found before for *F. japonica* (Mostaert et al. 1998;Marshall et al. 2002), *E. huxleyi* (Viso and Marty 1993;Bell and Pond 1996;Lang et al. 2011) and *T. weissflogii* (Viso and Marty 1993). In contrast, Lang et al. (2011) could not detect EPA as well as DHA in a *T. weissflogii* strain isolated from a brackish habitat. Although in general green algae do not contain high amounts of EPA and DHA, this is not the case for some marine *Chlorella* (Watanabe et al. 1983) and prasinophyte species (Viso and Marty 1993;Dunstan et al. 1992). In our study and in the study by Dunstan et al. (1992) relatively high amounts of DHA were found for the prasinophyte *Pyramimonas* sp.

Many earlier studies focused primarily on relative PUFA composition. Yet, Viso and Marty (1993) examined absolute fatty acid abundance and C/N ratios of 28 temperate marine microalgae from nine taxonomic classes, allowing the comparison of absolute PUFA content in terms of pg PUFA per cell. In our study EPA levels in the (cold) temperate species *T. weissflogii* varied between between 3.7 and 5.3 pg cell^-1^, while *E. huxleyi* contained a maximum DHA amount of 1.0 pg cell^-1^. Our levels were circa 10 times higher than EPA or DHA levels measured by Viso and Marty (1993) for these species (0.38 pg EPA cell^-1^ in *T. weissflogii* and 0.14 pg DHA cell^-1^ in *E. huxleyi*). This implies that the potential use of these species for future EPA/DHA production might be higher than earlier expected.

It has been postulated that microorganisms from polar regions contain relatively high (long chain) PUFA levels to maintain cell membrane fluidity (Nichols et al. 1993;Nishida and Murata 1996), but this was not confirmed by our study. For example, biovolume normalized EPA levels were higher in the polar diatom *C. brevis* than in the temperate diatom *T. weissfloggii,* whereas DHA levels were higher in the latter species. Also, no significant intraspecific effect of temperature on EPA or DHA content was found for the two investigated diatoms *C. brevis* and *T. weissflogii*. Thompson et al. (1992) studied the effect of temperature over the range from 10 to 25°C on fatty acid composition of eight species of marine phytoplankton, including *Thalassiosira pseudonana* and three species within the genus *Chaetoceros*. Pooled data from all species indicated a weak trend towards elevated PUFAs at lower temperature. However, only for *T. pseudonana* the percentage of the essential fatty acid DHA decreased linearly with increasing temperature. In addition, other studies (e.g. Teoh et al. 2004;Rousch 2003) showed varying results. Here a complicating factor could be that in many studies, including the present study, the distribution of PUFAs into the different lipid classes was not determined. Chen et al. (2008) suggested that low temperature could change the distribution of PUFAs in phospholipids, which contain high percentages of EPA and DHA, while at the same time not significantly affecting their total amount.

Since PUFAs play a role in the functioning of the thylakoid membrane, irradiance might theoretically affect (LC)-PUFA content in marine microalgae. In some studies light intensity indeed was negatively correlated with PUFA content (Thompson et al. 1990;Guedes et al. 2010;Leu et al. 2010), but the effect was often small, while sometimes no or a positive irradiance effect was found (e.g. Chrismadha and Borowitzka 1994;Tzovenis et al. 1997;Carvalho and Malcata 2005;Zhukova 2007;Guiheneuf et al. 2009). In our study no significant effect of irradiance on LC-PUFA content could be demonstrated for the two investigated diatoms *C. brevis* and *T. weissflogii*, while for both species chlorophyll *a* levels were clearly influenced by irradiance, indicating photoacclimation to the applied irradiance levels.

PUFA composition and production rate were found to be strongly species specific in our study. For example, the highest daily EPA production rate was found in the Antarctic diatom *Chaetoceros brevis*, while DHA production was highest in the cold-temperate prymnesiophyte *Emiliania huxleyi*. These results are consistent with earlier findings that EPA and DHA content differs systematically between taxonomic groups (Brown 2002). This implies that EPA and DHA productivity in algal mass cultures first of all benefits from thorough species selection: Mass cultivation at low temperature would benefit greatly from the relatively high growth rates exhibited by polar species at these low temperatures (*C. brevis*: 0.47 d^-1^ at 3°C, compared to *T. weissflogii* at 16°C: 0.49 d^-1^), while having similar LC-PUFA contents. Secondly, EPA and DHA production will gain primarily from the enhancement of growth rates or cell densities rather than from temperature or light-induced shifts in cellular EPA or DHA. EPA and DHA production rates determined in this study were lower than values reported elsewhere. For example, Meiser et al. (2004) cultured *Phaeodactylum tricornutum* in a flat panel airlift photobioreactor at high irradiance (1000 μmol photons m^-2^ s^-1^, 24 h day^-1^) and increased CO_2_ concentration resulting in a maximal EPA productivity of 118 mg L^-1^ day^-1^, which is circa 700 times higher than maximal productivity values from this study. Carvalho and Malcata (2005) reported a maximal productivity in *Pavlova lutheri* of 3.6 mg L^-1^ day^-1^ for EPA and 1.3 mg L^-1^ day^-1^ for DHA, which is circa 8 times higher than the DHA productivity values we determined for *E. huxleyi*. However, in our study we did not aim to optimize growth rates and cell densities for the individual species under study: our goal was to compare temperature and irradiance effects on PUFA composition in dilute cultures under standardized, nutrient replete conditions at sub-optimal temperatures.

We conclude that effective, low temperature mass cultivation of marine algae for EPA and DHA production would benefit from careful target species selection. Subsequently, for optimizing EPA/DHA yield merely specific growth rate needs to be considered rather than intracellular PUFA variability. In this respect, polar species cannot be ruled out *per se* since cold adapted algal species can exhibit high growth rates while at the same time synthesizing high EPA and/or DHA levels.

## Competing interests

The authors declare that they have no competing interests.
